# The Transport of the Territorial Medical Service

**Published:** 1909-12-18

**Authors:** 


### THE TRANSPORT OF THE TERRITORIAL MEDICAL SERVICE.

A new issue of the Establishments of the Territorial Force for 1909-1910 has just appeared, and in it are given the personnel of the different units and the scale of vehicles to be issued to them both for instructional purposes and for training in camp. According to it, the transport personnel of a Mounted Brigade Field Ambulance is to number 33, and of an Infantry Field Ambulance 38 non-commissioned officers and men; but with neither of the units is there to be a special transport officer. The transport duties are to be performed by one of the medical officers on the strength of the unit. For the training of this transport personnel the regulations lay it down that each non-commissioned officer and man must first put in 42 drills in First Aid and Ambulance work. Having done this he must attend 15 mounted drills per year, of which 7 must be riding ones in the school and 8 driving ones outside, either as postilion or with long rein driving from the box of the wagon. For the General Hospitals no transport personnel is provided, on the ground, we surmise, that they are regarded as stationary and that all their needs in connection with the Clearing Hospitals from whom they receive their sick and wounded will be met by the Army Service Corps.

Of the transport vehicles allowed for each medical unit a Mounted Brigade Field Ambulance has two heavy 4-horse ambulance wagons Mark V*, two light 2-horse ambulance wagons Mark I, one 1-horse filter water-cart, one 1-horse hired civilian wagon for medical stores, and one 1-horse hired civilian cart, weighing 11 cwt., for baggage stores. The horses allowed for the above field medical unit are 16 draught horses and 9 riding horses, six of the latter being for officers and three for non-commissioned officers. For a Field Ambulance there are allowed three heavy 2-horse ambulance wagons Mark V*, one 1-horse filter water-cart, one 2-horse hired civilian wagon for medical stores, one 2-horse hired civilian wagon (weighing about 19 cwt.) for baggage, stores, and supplies, and one 1-horse hired civilian spring-cart of about 9 cwt., for medical stores. The regulation number of horses for this medical unit is 12 draught horses and 14 riding ones, 10 of the latter being for officers and four for non-commissioned officers. To none, however, of the medical units is there to be any issue of service harness or saddlery, according to these recent tables of equipment.

In giving the field medical units permanently attached transport instead of having it detailed from the Army Service Corps, as is done with other units, the military authorities are following a course of action that is imperatively called for and has the sanction and support of the experience furnished by the wars of the past. When it is remembered that these medical units have to transport all their medical stores, their baggage (including tentage), and also to remove the sick and wounded, it is not difficult to realise what failure on their part to carry out their duties means to the army in the field. In view of this it is essential that all medical transport should be highly efficient as to personnel, and be furnished with the best and latest vehicles that can be obtained. Judged by these standards, we are not satisfied that full justice is being done to the field medical units in this matter, and we consider it our duty to draw attention to the fact. As regards the personnel, no fault can be found with their numbers or with the qualifying course of instruction laid down. Exception, however, must be taken to the arrangement by which one of the medical officers of the Field Ambulance is to act as transport officer and be responsible not only for a knowledge of his ambulance duties, but for those also of the transport section of his unit. To make it a regulation that a medical officer is to act in this dual capacity is very inadvisable. What is needed is that each field medical unit should have on its strength an officer for transport duties only, and he should be possessed of the technical knowledge that would allow of his instructing the transport personnel of the unit in their work.

In the matter of the horses and technical vehicles supplied we observe that there is a very considerable reduction in both of them compared with what was allowed in the first issue of the Territorial Force Regulations. Under the latter the Mounted Brigade Field Ambulance had eight four-horse ambulance wagons, four heavy and four light, with two general service wagons and two forage carts, while a field ambulance had six 2-horse ambulance wagons, six general service wagons, and three forage carts. If the present allowance is sufficient for instructional and training purposes no fault can be found with the issue, but we notice with regret the withdrawal of service technical vehicles and the substitution of hired civilian transport material. Of this step we highly disapprove, as also of the withdrawal of all regulation harness. The authorities must know that none of the service ambulance wagons can be fitted with ordinary civilian harness. These vehicles are furnished with chain loops on the swingle-trees and require a special trace connection which is not used in civil life, so that in the absence of Government harness the vehicles are useless for instructional and training work. Such a serious deficiency must either be met by the County Associations buying the necessary harness, or by the authorities furnishing all that is needed. We believe that the latter course is under consideration, and it should be adopted without delay.

There is no branch of the Service that calls for efficient transport more than the medical units, looking to their importance to the Army in the field, and it is a short-sighted policy that would work economy in this particular department. To do so is not acting fairly to the officers and men who voluntarily come forward and give their services to the country, while it is not a real saving in the end. No units that are furnished with nondescript vehicles and unreliable harness can feel an interest in their work or carry out their duties efficiently, while men so trained cannot take their places in the ranks of the Royal Army Medical Corps Transport should the occasion arise. The Volunteer Force had scant justice done it by the authorities in the matter of equipment, and we hope that there is not going to be a return to a policy that is unfair to the Territorial Force and detrimental to the interests of the country. It is because we regard the present state of matters as serious that we draw attention to them, for a defective transport service in the case of field medical units is sure to seriously cripple their efficiency in the event of mobilisation for invasion. It was stated at its formation that the Territorial Medical Service would be modelled on that of the Regular Army. It is not so if it is differently equipped, and in no way could transport organised on the present lines be considered as reliable and trustworthy.

